# Causality between COVID-19 and multiple myeloma: a two-sample Mendelian randomization study and Bayesian co-localization

**DOI:** 10.1007/s10238-024-01299-y

**Published:** 2024-02-24

**Authors:** Shuaiyuan Wang, Na Zhao, Ting Luo, Songzi Kou, Miaomiao Sun, Kuisheng Chen

**Affiliations:** 1 Department of Pathology, The First Affiliated Hospital of Zhengzhou University, Zhengzhou, 450052 Henan China; 2 Academy of Medical Science, Zhengzhou University, Zhengzhou, 450052 Henan China; 3 Henan Key Laboratory of Tumor Pathology, Zhengzhou University, Zhengzhou, 450052 Henan China

**Keywords:** Multiple myeloma, COVID-19, Bayesian co-localization, Causality, Mendelian randomization

## Abstract

Infection is the leading cause of morbidity and mortality in patients with multiple myeloma (MM). Studying the relationship between different traits of Coronavirus 2019 (COVID-19) and MM is critical for the management and treatment of MM patients with COVID-19. But all the studies on the relationship so far were observational and the results were also contradictory. Using the latest publicly available COVID-19 genome-wide association studies (GWAS) data, we performed a bidirectional Mendelian randomization (MR) analysis of the causality between MM and different traits of COVID-19 (SARS-CoV-2 infection, COVID-19 hospitalization, and severe COVID-19) and use multi-trait analysis of GWAS(MTAG) to identify new associated SNPs in MM. We performed co-localization analysis to reveal potential causal pathways between diseases and over-representation enrichment analysis to find involved biological pathways. IVW results showed SARS-CoV-2 infection and COVID-19 hospitalization increased risk of MM. In the reverse analysis, the causal relationship was not found between MM for each of the different symptoms of COVID-19. Co-localization analysis identified LZTFL1, MUC4, OAS1, HLA-C, SLC22A31, FDX2, and MAPT as genes involved in COVID-19-mediated causation of MM. These genes were mainly related to immune function, glycosylation modifications and virus defense. Three novel MM-related SNPs were found through MTAG, which may regulate the expression of B3GNT6. This is the first study to use MR to explore the causality between different traits of COVID-19 and MM. The results of our two-way MR analysis found that SARS-CoV-2 infection and COVID-19 hospitalization increased the susceptibility of MM.

**Supplementary Information:**

The online version contains supplementary material available at 10.1007/s10238-024-01299-y.

## Introduction

Over the past four years, Coronavirus 2019 (COVID-19), caused by Severe Acute Respiratory Syndrome Coronavirus 2019 (SARS-CoV-19), has spread around the world and is causing great concern globally [38]. Reportedly, COVID-19 may be able to co-exist with humans for a long time. The clinical presentation of patients with COVID-19 is diverse, and a variety of complications are frequently observed in patients with COVID-19, including interstitial pneumonia, pancytopenia, arthralgia and autoimmune diseases [9, 15]. Conversely, autoimmune diseases and inflammation lead COVID-19 patients into a vicious cycle of infection, causing increased morbidity and mortality [27, 35]. Autoimmune diseases such as multiple myeloma (MM), systemic lupus erythematosus and multiple sclerosis are extremely similar to COVID-19 in terms of symptomatology and pathological response, suggesting that there may be a close association between the diseases. A causal relationship between systemic lupus erythematosus, multiple sclerosis and COVID-19 has been reported [34]. However, there is still a gap in the causal relationship between MM and COVID-19. High doses of corticosteroids are used in the treatment of MM, and this usually leads to immunosuppression and a high inflammatory response, increasing the severity of COVID-19 [14]. A number of studies have reported, People with pre-existing autoimmune diseases are at higher risk of SARS-CoV-19 infection and disease severity [12]. Many other studies have observed that MM patients with COVID-19 have rates of infection and severe disease that are within the range of the general population [4]. Causal inferences from observational studies are unreliable or even contradictory due to the effects of unmeasured or unknown confounding factors [7]. On the other hand, due to ethics, it was impossible to conduct randomized controlled trial (RCTs) to investigate the causal relationship between COVID-19 and MM. Thus, the causal relationship between COVID-19 and MM remains ambiguous.

Clarifying the relationship between different traits of COVID-19 and MM helps in the diagnosis, treatment and rehabilitation of MM patients with COVID-19, assess the feasibility and risks of vaccination for patients with MM and help to develop strategies for management of patients with MM and for COVID-19 prophylaxis. Therefore, it is necessary to investigate the causal relationship between COVID-19 and MM [39].

Mendelian randomization (MR) is based on Mendel’s laws of inheritance, and use single nucleotide polymorphisms (SNPs) as an instrumental variable to assess the causal relationship between exposure factors and related outcomes [19]. Because genetic variation has occurred before the outcome, MR reduces the confounding effect of environmental factors and demonstrates genetic causality between exposure and outcome [19].

To fill this gap, we performed a bidirectional two-sample MR analysis to assess the causal relationship between different traits of COVID-19 and MM. LDSC calculates heritability, MTAG identifies novel possible causal SNPs, and combined analysis of co-localization and over-representation enrichment analysis reveal possible biological pathways for COVID-19 to cause MM. This facilitates a deeper understanding of the complex molecular mechanisms behind COVID-19 and MM.

## Methods

### Study design

**Fig. 1:**
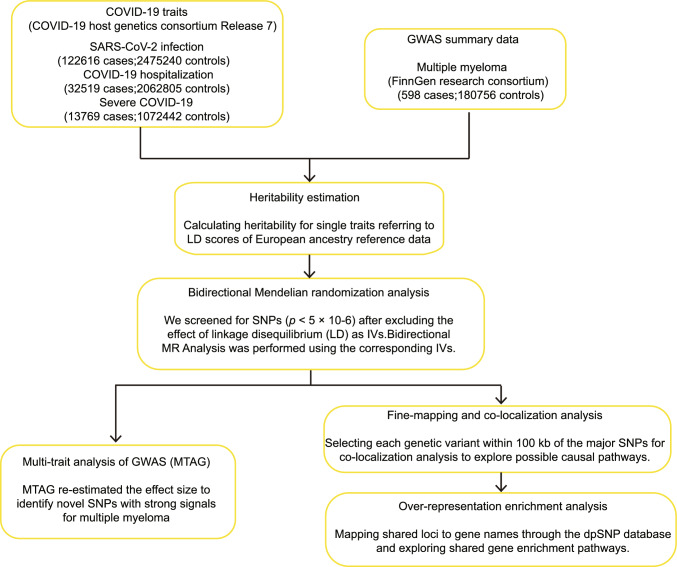
Flowchart of overall study design. GWAS, genome-wide association study; IVs, instrumental variables; SNPs, single nucleotide polymorphisms

**Fig. 2:**
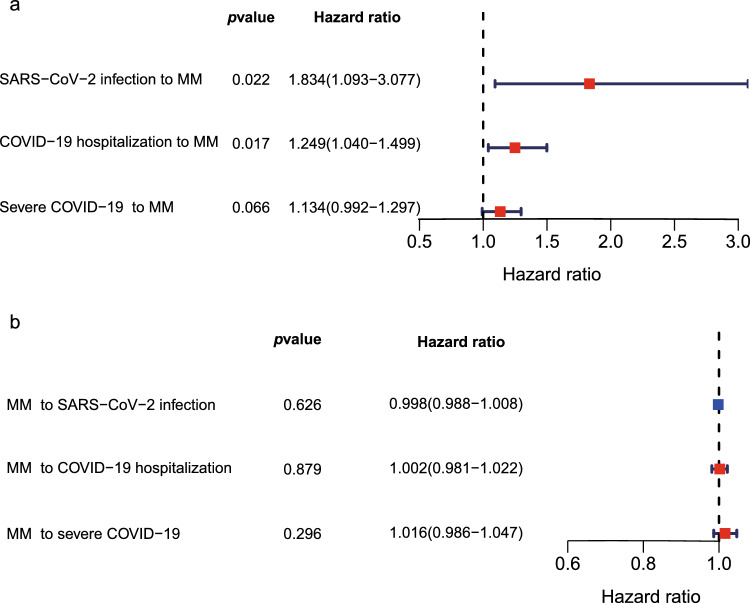
Bidirectional Mendelian randomization analysis using inverse-variance weighting (IVW) method. **a** Coronavirus Disease (COVID-19) related traits to multiple myeloma (MM); **b** multiple myeloma (MM) to Coronavirus Disease (COVID-19) related traits

**Fig. 3:**
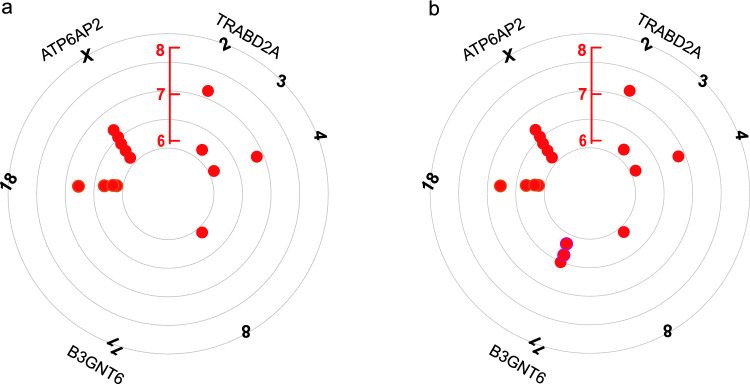
Circular Manhattan plot of significant SNP correlation with multiple myeloma (MM). **a** The significant SNP correlation with MM before MTAG analysis; **b** the significant SNP correlation with MM after MTAG analysis

**Fig. 4:**
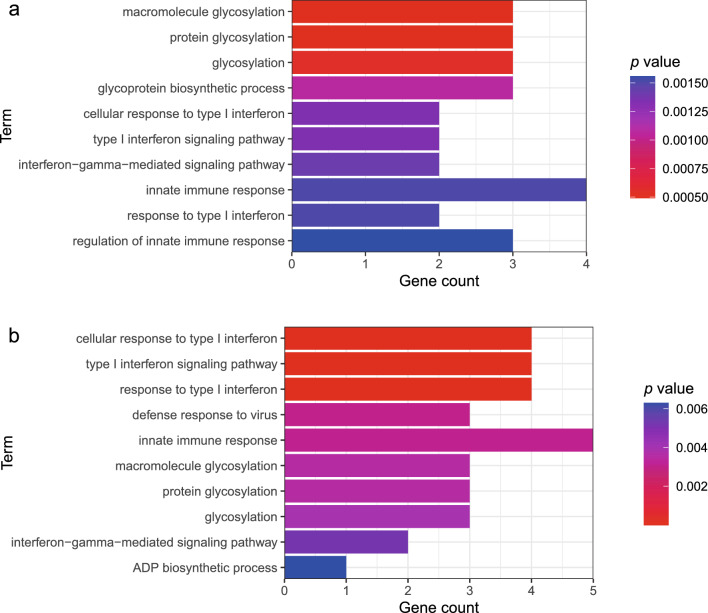
Over-representation enrichment analysis of shared genes. **a** The shared genes of SARS-CoV-2 infection and MM. **b** The shared genes of COVID-19 hospitalization and MM

The overall study design is shown in Fig. 1. Our study is based on multiple GWAS studies. We firstly calculate heritability of COVID-19 related traits (SARS-CoV-2 infection, COVID-19 hospitalization and severe COVID-19) and MM to show that they are all hereditable. Then selecting instrumental variables (IVs), we used bidirectional MR analysis [19] to infer the causal relationships between COVID-19 related traits and MM. Based on the results of bidirectional MR analysis, we expect to identify more novel SNPs with strong signals for MM using multi-trait analysis of GWAS (MTAG) [30] incorporating information from GWAS of COVID-19. In addition, we performed fine-mapping and co-localization analysis [16] to identify shared loci between COVID-19 and MM. We undertook over-representation enrichment analysis [31] of the shared loci to explore the biological processes involved.

### Data sources

Genetic associations of COVID-19 were obtained from the latest freeze 7 data (release date: April 8, 2022) based on European ancestry—populations of the COVID-19 host genetics Initiative (COVID-19 HGI) (https://www.covid19hg.org/results/covidhgi-freeze-7-readme.txt) [21]. The data contain three relevant features of COVID-19: (1) Individuals with very severe respiratory symptoms confirmed by COVID-19 or who died from the disease are called severe COVID-19 (13,769 cases and 1,072,442 controls). (2) Individuals hospitalized for symptoms of associated infections are called COVID-19 hospitalization (32,519 cases and 2,062,805 controls). (3) All individuals reporting positive SARS-CoV2 infection, regardless of symptoms and hospitalization, are called SARS-CoV2 infection (122,616 cases and 2,475,240 controls). The genome-wide association studies (GWAS) data for MM patients from the FinnGen GWAS database [10], comprising 598 patients and 180,756 controls of European ancestry. The data in the two databases are not raw genotyping data, but summary statistics data after strict and standardized quality control. Therefore, there is no need for quality control again.

### Linkage disequilibrium score regression (LDSC)

Referring to LD scores of European ancestry reference data from the 1000 Genomes Project, we used GWAS summary statistics to calculate heritability for single traits. The LDSC provided an estimate of heritability by regressing the LD score on the running test statistic [8]. LDSC can provide accurate estimates, even if test statistics are inflated due to polygenicity [33]. The heritability estimation was completed by the “ldsc” R software package.

### MR analysis

We screened for SNPs that reached the genomic significance level (*p* < 5 $\times$ 10 ^-6^). Secondly, to exclude the effect of linkage disequilibrium (LD) on the MR results, we used the clumping process (*r*^2^ < 0.001, clumping distance = 10,000 kb) to ensure that there is no LD between SNPs. Finally, selected SNPs were matched with the GWAS of outcome, and the missing SNPs were replaced by SNPS with high LD (*r*^2^ > 0.8). Finally, after removing palindromic SNPs, the other SNPs were used as IVs.

“TwoSampleMR” R package (version 0.5.6, Stephen Burgess, Chicago, IL, USA) was used for two-sample MR Analysis between exposure and outcome. The bidirectional MR analysis is an MR analysis that tries to differentiate whether factor *A* is a cause or a consequence of factor *B* or whether there is a true bidirectional causal effect between *A* and *B*. Inverse variance weighting (IVW, random effects) was used as the main method of analysis. Cochran’s *Q* statistic was adopted for IVW analysis to detect the heterogeneity, and *p* > 0.05 indicates no heterogeneity [28]. MR-Egger regression was used to identify potential pleiotropy and to assess the effect of pleiotropy on the risk assessment of the intercept test, with *p* > 0.05 indicating no pleiotropy [36].

### MTAG

The key assumption of MTAG is that all SNPs share the same variance-covariance matrix of effect sizes across traits. MTAG re-estimated the effect size for each trait by combining the weighted sum of the GWAS estimates for different traits to identify novel SNPs with strong signals [30]. Compared with genome-wide significant SNPs identified in single-trait GWAS, more phenotype-related SNPs can be found through joint analysis of multiple traits related GWAS summary results. We used trait-specific effective sample sizes and transformed *Z*-scores for MTAG. All SNPs present in the GWAS data were included in the MTAG calculation.

### Fine-mapping and co-localization analysis

The “COLOC” R package was used to assess the probability that two traits share the same causal variant. We selected each genetic variant within 100 kb of the major SNPs for co-localization analysis [13]. By calculating the posterior probability that two traits share the same causal variant (*p*(H4)), we explore possible causal pathways by which COVID-19 causes MM. If any SNP has a *p* (H4) > 0.85, we label it as a co-localized genetic variant [16].

### Over-representation enrichment analysis

We mapped shared loci to gene names through the dpSNP database. Enrichment in Gene Ontology (GO) biological process was analyzed by the WEB-based Gene Set Analysis Toolkit using genes with *p*(H4) > 0.8 [31]. We performed over-representation enrichment analysis for shared genes of SARS-CoV-2 infection and MM, COVID-19 hospitalization and MM in order to explore the biological processes involved in the causal relationship between COVID-19 and MM.

## Results

### Bidirectional MR analysis

Heritability (h2) estimated that all three traits of COVID-19 (severe COVID-19, COVID-19 hospitalization, SARS-CoV-2 infection) and MM were heritable (*p* < 0.05, as shown in Table 1). We next performed MR analysis of COVID-19 to MM (Fig. 2a). The results of IVW suggested SARS-CoV-2 infection had a positive effect on MM (*p* = 0.021), and COVID-19 hospitalization also increased the risk of MM (*p* = 0.017). Heterogeneity test and MR-Egger intercept test did not demonstrate evidence of heterogeneity or horizontal pleiotropy (Table 2), which supported the validity of the findings. The causal effect of severe COVID-19 to MM was not observed (*p* = 0.066).

**Table 1:** SNP based heritability estimated by LDSC

Trait	Heritability (h2)	Heritability SE	Heritability *P*
SARS-CoV-2 infection	0.045049895	0.006941053	8.56E$-$11
COVID-19 hospitalization	0.264955035	0.031788277	7.75E$-$17
Severe COVID-19	0.476456733	0.074659044	1.75E$-$10
Multiple myeloma	0.005518633	0.000288947	2.57E$-$81

**Table 2:** The corresponding pleiotropy and heterogeneity test of each exposure-outcome pair

Exposure	Outcome	Intercept	*P*-value of intercept	*Q* Statistic	*P*-value of *Q* statistic
SARS-CoV-2 infection	MM	0.013721	0.5276679	48.1695	0.4253095
COVID-19 hospitalization	MM	$-$ 0.01647	0.2936542	78.33976	0.5316208

**Table 3:** Significant SNPs of MM after MTAG analysis

SNP	CHR	A1	A2	Representative gene	BETA	SEBETA	*P* value
rs59567236	2	G	A	TRABD2A	5.683	1.1149	3.44E$-$07
rs765309482	3	A	G	RBMS3	46.8411	10.2106	4.49E$-$06
rs75358643	4	G	A	FSTL5	1.75	0.333	1.48E$-$07
rs559179871	4	T	C	ANXA10	4.1188	0.9016	4.91E$-$06
rs78782621	8	C	T	TRPS1	1.2687	0.2759	4.27E$-$06
rs61034889	11	A	G	B3GNT6	0.7428	0.1633	5.42E$-$06
rs59762951	11	A	G	B3GNT6	0.7428	0.1633	5.42E$-$06
rs73493619	11	A	G	B3GNT6	0.7429	0.1633	5.41E$-$06
rs747301490	18	A	G	SALL3	20.5039	4.1584	8.19E$-$07
rs1039900397	18	G	C	ATP9B	18.9888	4.015	2.25E$-$06
rs970051324	18	G	C	ATP9B	19.1489	3.9656	1.37E$-$06
rs554481279	18	G	A	NFATC1	17.8689	3.7534	1.93E$-$06
rs115162022	X	T	A	ATP6AP2	0.9453	0.2038	3.52E$-$06
rs17314654	X	T	C	ATP6AP2	0.945	0.2056	4.33E$-$06
rs116677033	X	C	T	ATP6AP2	0.9271	0.1956	2.13E$-$06
rs145079441	X	T	C	ATP6AP2	0.926	0.193	1.61E$-$06
rs142284554	X	C	G	ATP6AP2	0.9179	0.1923	1.81E$-$06

CHR, chromosome; POS, position; A1, effect allele; A2, other allele; SEBETA, standard error of BETA

**Table 4:** Co-localization analysis of COVID-19 and MM

SNP	CHR	POS	A1	A2	*p*(H4)	Representative gene
*SARS-CoV-2 infection to MM*						
rs73062389	3	45,793,925	A	G	0.8949859	SLC6A20
rs35044562	3	45,867,532	G	A	0.8949855	LZTFL1
rs2260685	3	195,770,872	C	T	0.8666474	MUC4
rs10774673	12	112,923,353	T	C	0.8658866	OAS1
rs9264740	6	31,276,554	T	C	0.8535353	HLA-C
*COVID-19 hospitalization to MM*						
rs12585036	13	113,535,741	T	C	0.9617841	ATP11A
rs4475253	5	131,776,506	G	A	0.9615472	IRF1-AS1
rs63750417	17	44,060,775	T	C	0.9241245	MAPT
rs189201949	19	43,664,483	C	G	0.9202383	PSG5
rs117169628	16	89,262,657	A	G	0.9116342	SLC22A31
rs78314212	21	35,312,916	T	C	0.9048163	LINC00649
rs41435745	6	41,490,382	C	G	0.9009399	FOXP4
rs67959919	3	45,830,416	A	G	0.896943	LZTFL1
rs11654648	17	47,836,287	T	C	0.8969175	LRRC46
rs17279437	3	45,772,602	A	G	0.896888	SLC6A20
rs41264915	1	155,197,995	G	A	0.8930309	THBS3
rs1498399	1	77,481,554	G	A	0.8921093	AK5
rs78295726	19	10,315,836	T	C	0.8920472	RAVER1,FDX2
rs45524632	19	10,486,312	A	C	0.8879368	KEAP1
rs676314	19	50,362,278	G	A	0.8847234	NAPSA,NR1H2
rs35705950	11	1,219,991	T	G	0.8821934	MUC5B
rs8192330	8	22,163,617	A	G	0.871739	SFTPC
rs77566758	1	154,865,312	T	C	0.8707566	KCNN3
rs139589338	1	154,853,813	G	A	0.870755	KCNN3
rs4767025	12	112,920,989	T	C	0.8679668	OAS1
rs58572235	5	29,837,133	T	C	0.8670273	NA
rs1123573	2	60,480,453	G	A	0.8547268	BCL11A

CHR, chromosome; POS, position; A1, effect allele; A2, other allele

The results of MR Analysis of MM to COVID-19 are shown in Fig. 2b. We found null causal relationship of genetically predicted MM to severe COVID-19 (*p* = 0.296), COVID-19 hospitalization (*p* = 0.879) and SARS-CoV-2 infection (*p* = 0.626). IVs used in MR analysis for individual traits are shown in supplemental Tables 1–4.

### MTAG

Fourteen genomic significant and independent genetic loci for MM were extracted using GWAS of European ancestry (Fig. 3a). We performed a joint GWAS using MTAG for two COVID-19 traits (SARS-CoV-2 infection, COVID-19 hospitalization) with MM. Finally, a total of 17 significant loci associated with MM were identified (Table 3). We observed that there were three more relevant loci than those identified by single-trait GWAS. Interestingly, all three loci are located on chromosome 11 and may regulate the expression of B3GNT6 (Fig. 3b). This gene encodes B3GNT6 protein, the absence of which predisposes to the development of chronic inflammation and ultimately to the development of cancer. In addition, we observed that three SNPs strongly associated with MM were located on the X chromosome, which may be one of the reasons why there are more males than females in patients with MM. We did not identify other genome-wide significant loci for the COVID-19 trait using MTAG combining information from GWAS associated with MM.

### Analysis of shared genetic loci

Although we have found evidence of a positive causal effect of COVID-19 on MM, the causal mechanisms by which COVID-19 promotes MM remain to be explored. We defined each individual SNP and genetic variation within 100kb associated with COVID-19 or MM as a test region. Finally, after merging the overlapping regions, a total of 53 unique regions of SARS-CoV-2 infection and MM were included, and a total of 86 unique regions of COVID-19 hospitalization and MM were included.

Among these unique regions tested, five regions in chromosomes 3, 6, and 12 suggested causal SNPs of SARS-CoV-2 infection on MM (co-localization probability *p*(H4) > 0.85; Table 4). There are three regions on chromosome 3 and one region on each of chromosomes 6 and 12. The co-localized genetic loci were mapped to SLC6A20, LZTFL1, MUC4, OAS1, HLA-C genes for MM with SARS-CoV-2 infection (Table 4). In addition to these chromosomes, the causal SNPS of COVID-19 hospitalization on MM are also located in many regions of chromosome 19, involving genes such as PSG5, SLC22A31, FDX2, MAPT and others. ABO and FUT2 all contribute to both autoimmune diseases and COVID-19. Chromosome 6 has a region of the human leukocyte antigen (HLA) complex that is strongly associated with immunity and encodes a variety of cell surface proteins responsible for regulating the immune system, including HLA-C. Autoimmune diseases have also been repeatedly reported to be associated with genes on chromosomes 3 and 19 [18]. Combining MR results showing causal effects of SARS-CoV-2 infection and COVID-19 hospitalization to MM and no evidence of horizontal pleiotropy, these observed genes may exert causal effects on MM through changes in SARS-CoV-2 infection and COVID-19 hospitalization.

### Over-representation enrichment analysis

In over-representation enrichment analysis, these shared genes were enriched in several immune response-related pathways (Fig. 4), such as “response to type I interferon”, “type I interferon signaling pathway”, “interferon-gamma-mediated signaling pathway”, “glycosylation of proteins and macromolecules”, “innate immune response” and “defense response to virus”. These biological processes are closely related and important for COVID-19 and MM.

## Discussion

The malignant plasma cell neoplasm is a hematological disorders caused by clonal proliferation of plasma cells and the resulting overproduction of monoclonal immunoglobulins (M proteins). MM is an important category. MM patients exhibit various immune deficiencies caused by the disease or its treatment [3]. There are also complex interactions between SARS-CoV-2 and the immune system [22]. It is important to determine whether there is a potential relationship between COVID-19 and MM and to understand the complex molecular mechanisms behind them. This contributes to a deeper understanding of the relationship between COVID-19 and MM and provides insights into future viral crisis events similar to SARS or SARS-CoV-2. To our knowledge, this report is the first to provide evidence for the presumed causal nature of the association between COVID-19 and MM.

Autoantibody production is a key feature of autoimmune diseases, and autoantibodies known to occur in many autoimmune diseases have been detected in patients with COVID-19 [17]. SARS-CoV-2 can disrupt self-tolerance and trigger an autoimmune response through cross-reactivity with host cells [2]. Some patients have been reported to develop autoimmune diseases, such as Guillain-Barre syndrome or systemic lupus erythematosus, after contracting COVID-19 [6]. Our MR study suggests that SARS-CoV-2 infection and COVID-19 hospitalization are associated with an increased risk of MM. By using a bidirectional analysis strategy, we can distinguish between upstream and downstream of the disease chain without reverse causation. We show for the first time that SARS-CoV-2 infection and COVID-19 hospitalization are associated with a higher risk of MM genetically. Our results support the conclusion that COVID-19 may trigger autoimmunity and induce autoimmune diseases [24].

When the GWAS of a phenotype is correlated with the results of other phenotypes, MTAG can use the information of the correlated phenotypes to conduct joint multi-trait analyses to identify novel SNPs of significance. We observed three novel SNPs, which coincidentally clustered around B3GNT6. B3GNT6, which encodes an important precursor in the biosynthesis of mucin-type glycoproteins, is associated with autoimmune diseases and DNA repair, such as selective lgA deficiency [23].

We applied an integrated approach combining MR with COLOC to investigate the genetic causal pathway of MM mediated by SARS-CoV-2 infection and COVID-19 hospitalization. MR + COLOC analysis showed evidence of causal effects of five genes mediated by SARS-CoV-2 infection and 19 genes mediated by COVID-19 hospitalization on MM. Some of these genes have been reported to be associated with COVID-19 and may be responsible for increased susceptibility to autoimmune diseases. MUC4 controls ciliary motility and contributes in defensing against allergens, viruses and extracellular molecules [25]. What is more, MUC4 is highly associated with SARS-CoV-2 infection, and its subsequent dysregulated immune-inflammatory response plays an important role in the pathogenic mechanisms of autoimmune diseases such as rheumatoid arthritis and systemic lupus erythematosus [29]. The HLA region contains genes involved in antigen processing, presentation, and immune regulation, and has a role in both COVID-19 and autoimmune diseases. HLA-C in the HLA I region is an allele closely associated with SARS-CoV-2 infection and also mediates a variety of autoimmune diseases [32]. There are also SLC6A20 [1] and LZTFL1 [11]. Variants within the ATP11A gene influence the development of illness requiring hospitalization after infection with SARS-CoV-2 [37]. Hu et al. [20] reported that low-expressed/highly methylated ATP11A is a prognostic marker for acute myeloid leukemia. These genes we identified are involved in three main biological processes: response to interferon, regulation of immune processes and glycosylation of proteins. It has been shown that genetic variation of functional genes in IFN-I pathway is associated with disease risk. Inhibition of IFN-I response can aggravate virus replication and lead to excessive immunity and induce a variety of immune diseases such as rheumatoid arthritis and systemic lupus erythematosus [26]. COVID-19-associated changes in levels and N-glycosylation of specific plasma proteins highlight the role and complexity of glycosylation in COVID-19 [5]. It has also been widely reported that glycosylation of immunoglobulins induced pathological changes and ultimately lead to the development of autoimmune diseases [40]. The glycosylation pathway may play a role in the causal relationship between COVID-19 and MM. Thus, these genes we identified may play important roles in the underlying mechanisms of MM, of which COVID-19 is the mediator.

Our study is the first to explore the relationship between COVID-19 related traits and MM. However, we acknowledge some limitations of our study. Firstly, we used GWAS data of European ancestor to avoid population stratification, so the applicability of our results to other populations (such as Asians) should be carefully considered. Secondly, we indirectly highlighted causal genes and pathways, and these findings require further confirmatory analyses. And finally, we did not find a causal relationship between severe COVID-19 and MM for reasons, so further observations and biological experiments may be needed.

## Conclusion

In summary, we report a causal relationship between COVID-19 and MM, identify possible causal pathways using an MR + COLOC analysis method, and discover the biological processes at work. These findings may provide new insights for further studies on the potential pathogenesis and therapeutic targets of MM.

## Supplementary Information

Below is the link to the electronic supplementary material.

Supplementary file 1 (docx 18 KB)

Supplementary file 2 (docx 21 KB)

Supplementary file 3 (docx 20 KB)

Supplementary file 4 (docx 14 KB)

## Data Availability

Genetic associations with COVID-19 and MM were obtained from the COVID-19 host genetics initiative (https://www.covid19hg.org/results/covidhgi-freeze-7-readme.txt) and FinnGen GWAS database(https://www.finngen.fi/en).

## Code availability

Code is available from the author on reasonable request.
