# Supplementary material for: Causality between COVID-19 and multiple myeloma: a two-sample Mendelian randomization study and Bayesian co-localization
Source: Clin Exp Med. 2024 Feb 24;24(1):42. doi: 10.1007/s10238-024-01299-y (PMC10894079; doi:10.1007/s10238-024-01299-y)
Supplement: Supplementary file 1 — (docx 18 KB) [file 10238_2024_1299_MOESM1_ESM.docx]

Supplementary Table 1. IVs we selected for SARS-CoV-2 infection.

| SNP | CHR | POS | ALT | REF | BETA | SE | *P* value |
| --- | --- | --- | --- | --- | --- | --- | --- |
| rs552414 | 1 | 65482060 | T | G | 0.035516 | 0.007355 | 1.37E-06 |
| rs76717728 | 1 | 82110334 | G | A | -0.05175 | 0.011224 | 4.01E-06 |
| rs138015863 | 1 | 101542485 | T | C | 0.09447 | 0.019077 | 7.34E-07 |
| rs1218556 | 1 | 154821134 | T | G | 0.064838 | 0.013803 | 2.63E-06 |
| rs11264339 | 1 | 155140648 | T | C | -0.03502 | 0.004562 | 1.62E-14 |
| rs145355387 | 1 | 181820465 | A | G | -0.07232 | 0.015356 | 2.49E-06 |
| rs1318827 | 1 | 218813701 | G | A | 0.024438 | 0.005044 | 1.26E-06 |
| rs13411576 | 2 | 983008 | C | T | 0.022894 | 0.00498 | 4.28E-06 |
| rs4665221 | 2 | 23772331 | C | T | 0.04895 | 0.010112 | 1.29E-06 |
| rs1123573 | 2 | 60707588 | G | A | -0.02639 | 0.004769 | 3.16E-08 |
| rs73062389 | 3 | 45835417 | A | G | 0.20044 | 0.009861 | 7.61E-92 |
| rs35044562 | 3 | 45909024 | G | A | 0.12798 | 0.008476 | 1.64E-51 |
| rs2290859 | 3 | 101525625 | T | C | -0.05084 | 0.00486 | 1.32E-25 |
| rs17584100 | 3 | 169631240 | T | C | -0.07351 | 0.014173 | 2.14E-07 |
| rs2260685 | 3 | 195497743 | C | T | 0.033425 | 0.004689 | 1.01E-12 |
| rs13107325 | 4 | 103188709 | T | C | 0.048238 | 0.008857 | 5.14E-08 |
| rs1479596 | 5 | 76552878 | G | A | -0.02125 | 0.004605 | 3.94E-06 |
| rs11950133 | 5 | 137995130 | G | T | 0.033513 | 0.006982 | 1.59E-06 |
| rs9264740 | 6 | 31244331 | T | C | -0.03047 | 0.00526 | 6.87E-09 |
| rs2068205 | 6 | 33058583 | C | T | 0.021454 | 0.004651 | 3.96E-06 |
| rs71561432 | 6 | 76243730 | G | T | 0.097504 | 0.020817 | 2.82E-06 |
| rs4723810 | 7 | 38962722 | G | C | -0.0347 | 0.007565 | 4.51E-06 |
| rs62482222 | 7 | 100197866 | A | G | -0.03525 | 0.006796 | 2.14E-07 |
| rs11143138 | 9 | 71228859 | T | G | 0.024801 | 0.005378 | 3.99E-06 |
| rs10759670 | 9 | 116753379 | G | A | 0.024936 | 0.004968 | 5.20E-07 |
| rs554833 | 9 | 136147160 | T | C | 0.090799 | 0.004765 | 5.87E-81 |
| rs721917 | 10 | 81706324 | G | A | 0.023043 | 0.004597 | 5.36E-07 |
| rs7118388 | 11 | 34454147 | G | A | 0.026551 | 0.004533 | 4.71E-09 |
| rs142669125 | 11 | 114741564 | A | G | 0.090838 | 0.019572 | 3.47E-06 |
| rs117498337 | 11 | 123076792 | G | C | 0.060365 | 0.012782 | 2.33E-06 |
| rs10774673 | 12 | 113361158 | T | C | 0.028916 | 0.00483 | 2.14E-09 |
| rs7973123 | 12 | 133136791 | T | G | 0.028891 | 0.005439 | 1.09E-07 |
| rs7318364 | 13 | 24772892 | T | C | 0.028871 | 0.006161 | 2.78E-06 |
| rs71436552 | 13 | 80723267 | A | C | -0.05459 | 0.011492 | 2.04E-06 |
| rs9589601 | 13 | 93257339 | G | A | 0.023471 | 0.005047 | 3.31E-06 |
| rs225580 | 13 | 105458374 | C | T | -0.02541 | 0.005501 | 3.86E-06 |
| rs184781326 | 14 | 29459234 | G | A | -0.06902 | 0.011679 | 3.42E-09 |
| rs116984801 | 14 | 100138404 | T | A | 0.084656 | 0.018065 | 2.78E-06 |
| rs184931682 | 16 | 48534793 | C | T | 0.069379 | 0.014977 | 3.62E-06 |
| rs79558487 | 16 | 75523406 | A | C | -0.05751 | 0.011565 | 6.61E-07 |
| rs12602513 | 17 | 38159744 | G | A | 0.023184 | 0.004903 | 2.26E-06 |
| rs16967171 | 18 | 20011523 | A | G | 0.027404 | 0.005839 | 2.68E-06 |
| rs12610495 | 19 | 4717672 | G | A | 0.049515 | 0.005077 | 1.80E-22 |
| rs73005873 | 19 | 9007630 | A | G | 0.02735 | 0.00508 | 7.27E-08 |
| rs280519 | 19 | 10472933 | G | A | 0.023047 | 0.004694 | 9.10E-07 |
| rs679574 | 19 | 49206108 | G | C | -0.03605 | 0.004596 | 4.37E-15 |
| rs676314 | 19 | 50865535 | G | A | 0.027577 | 0.004815 | 1.02E-08 |
| rs74947155 | 19 | 57733871 | A | G | 0.17034 | 0.036944 | 4.01E-06 |
| rs2834158 | 21 | 34617213 | C | T | -0.04055 | 0.004871 | 8.51E-17 |
| rs75586969 | 21 | 35265459 | T | C | 0.042988 | 0.008419 | 3.29E-07 |
| rs7289512 | 22 | 42796914 | A | G | 0.024422 | 0.004619 | 1.24E-07 |

Abbreviations: CHR, chromosome; ALT, effect allele; REF, other allele; SE, sebeta.
