# Supplementary material for: Causality between COVID-19 and multiple myeloma: a two-sample Mendelian randomization study and Bayesian co-localization
Source: Clin Exp Med. 2024 Feb 24;24(1):42. doi: 10.1007/s10238-024-01299-y (PMC10894079; doi:10.1007/s10238-024-01299-y)
Supplement: Supplementary file 2 — (docx 21 KB) [file 10238_2024_1299_MOESM2_ESM.docx]

Supplementary Table 2. IVs we selected for COVID-19 hospitalization.

| SNP | CHR | POS | ALT | REF | BETA | SE | *P* value |
| --- | --- | --- | --- | --- | --- | --- | --- |
| rs2297812 | 1 | 47276728 | C | G | -0.0492 | 0.01015 | 1.26E-06 |
| rs3014983 | 1 | 65460691 | T | C | -0.06951 | 0.012003 | 6.99E-09 |
| rs1498399 | 1 | 77947239 | G | A | 0.068773 | 0.010063 | 8.24E-12 |
| rs115545251 | 1 | 93042753 | A | G | 0.14576 | 0.028087 | 2.10E-07 |
| rs139589338 | 1 | 154826289 | G | A | 0.20902 | 0.03574 | 4.97E-09 |
| rs77566758 | 1 | 154837788 | T | C | 0.18818 | 0.040523 | 3.42E-06 |
| rs41264915 | 1 | 155167786 | G | A | -0.14296 | 0.015375 | 1.43E-20 |
| rs2473831 | 1 | 178456811 | G | A | 0.059545 | 0.012862 | 3.66E-06 |
| rs7532924 | 1 | 228393929 | A | C | 0.049893 | 0.009709 | 2.76E-07 |
| rs4952111 | 2 | 30443993 | T | C | 0.05213 | 0.011193 | 3.20E-06 |
| rs6732083 | 2 | 39081734 | C | G | -0.05346 | 0.011259 | 2.05E-06 |
| rs1123573 | 2 | 60707588 | G | A | -0.06885 | 0.010432 | 4.13E-11 |
| rs6730279 | 2 | 88556133 | C | T | -0.06827 | 0.014648 | 3.15E-06 |
| rs13018756 | 2 | 137008235 | T | C | -0.04758 | 0.010096 | 2.44E-06 |
| rs4146377 | 2 | 152848504 | T | C | 0.18402 | 0.037629 | 1.01E-06 |
| rs4439973 | 2 | 166322550 | A | G | -0.05478 | 0.011385 | 1.50E-06 |
| rs17006525 | 3 | 20115244 | C | T | -0.05618 | 0.012157 | 3.82E-06 |
| rs17279437 | 3 | 45814094 | A | G | -0.11372 | 0.016819 | 1.37E-11 |
| rs67959919 | 3 | 45871908 | A | G | 0.49158 | 0.017513 | 2.36E-173 |
| rs17412601 | 3 | 101499275 | C | T | -0.06826 | 0.010317 | 3.69E-11 |
| rs343318 | 3 | 146235991 | C | T | 0.093617 | 0.017513 | 9.02E-08 |
| rs11927731 | 3 | 169027826 | A | G | 0.077075 | 0.016275 | 2.18E-06 |
| rs7664615 | 4 | 25448493 | G | A | -0.07259 | 0.013409 | 6.18E-08 |
| rs34712979 | 4 | 106819053 | A | G | -0.06431 | 0.011488 | 2.17E-08 |
| rs143556778 | 4 | 108729685 | T | C | 0.12948 | 0.024986 | 2.19E-07 |
| rs13170352 | 5 | 718620 | T | C | 0.28006 | 0.061033 | 4.46E-06 |
| rs58572235 | 5 | 29837133 | T | C | 0.19915 | 0.037334 | 9.59E-08 |
| rs7706444 | 5 | 35644621 | C | T | 0.050841 | 0.010685 | 1.95E-06 |
| rs182192737 | 5 | 126917687 | T | C | -0.25009 | 0.053803 | 3.35E-06 |
| rs4475253 | 5 | 131776506 | G | A | 0.058194 | 0.010052 | 7.07E-09 |
| rs10070885 | 5 | 142020380 | T | C | -0.05623 | 0.01216 | 3.76E-06 |
| rs1634761 | 6 | 31274027 | T | C | -0.06768 | 0.009495 | 1.02E-12 |
| rs2068205 | 6 | 33058583 | C | T | 0.049815 | 0.0097 | 2.81E-07 |
| rs41435745 | 6 | 41490382 | C | G | 0.21307 | 0.035222 | 1.45E-09 |
| rs6901756 | 6 | 41825590 | C | T | -0.07444 | 0.014634 | 3.65E-07 |
| rs34138876 | 7 | 7067000 | C | T | 0.098238 | 0.020467 | 1.59E-06 |
| rs3757447 | 7 | 22894735 | T | A | -0.04849 | 0.009782 | 7.14E-07 |
| rs2897075 | 7 | 99630342 | T | C | 0.058531 | 0.009857 | 2.88E-09 |
| rs8192330 | 8 | 22021130 | A | G | -0.11296 | 0.021045 | 7.98E-08 |
| rs2326562 | 8 | 61428200 | T | C | 0.053141 | 0.009736 | 4.81E-08 |
| rs66539629 | 8 | 131657153 | T | C | 0.060311 | 0.012892 | 2.89E-06 |
| rs77264633 | 9 | 15823918 | G | A | 0.092284 | 0.0177 | 1.85E-07 |
| rs149533170 | 9 | 21172825 | A | G | 0.28389 | 0.051106 | 2.78E-08 |
| rs11790730 | 9 | 33425785 | C | T | 0.066822 | 0.012805 | 1.80E-07 |
| rs996856 | 9 | 109487910 | A | G | 0.047226 | 0.009959 | 2.12E-06 |
| rs657152 | 9 | 136139265 | A | C | 0.10009 | 0.009764 | 1.18E-24 |
| rs7921998 | 10 | 30830248 | G | A | 0.10737 | 0.022946 | 2.88E-06 |
| rs17885848 | 10 | 81316456 | T | C | 0.061114 | 0.010877 | 1.93E-08 |
| rs35705950 | 11 | 1241221 | T | G | -0.09893 | 0.015532 | 1.90E-10 |
| rs61882275 | 11 | 34504292 | A | G | -0.09196 | 0.009945 | 2.30E-20 |
| rs7106973 | 11 | 130758113 | A | G | 0.047169 | 0.009618 | 9.38E-07 |
| rs4767025 | 12 | 113358794 | T | C | 0.07545 | 0.010079 | 7.13E-14 |
| rs5023077 | 12 | 133141973 | C | T | -0.06608 | 0.009515 | 3.79E-12 |
| rs12585036 | 13 | 113535741 | T | C | 0.096736 | 0.011638 | 9.39E-17 |
| rs4903661 | 14 | 78335939 | G | A | -0.04564 | 0.009667 | 2.35E-06 |
| rs117293456 | 14 | 98373829 | C | T | 0.14865 | 0.031149 | 1.82E-06 |
| rs10129899 | 14 | 104330432 | A | C | -0.0566 | 0.012214 | 3.59E-06 |
| rs2445772 | 15 | 51641996 | G | C | 0.052075 | 0.011104 | 2.74E-06 |
| rs12908340 | 15 | 64427704 | C | A | 0.059187 | 0.011763 | 4.86E-07 |
| rs78137607 | 16 | 3939922 | A | G | 0.24354 | 0.048687 | 5.67E-07 |
| rs2102497 | 16 | 54255222 | C | T | 0.064371 | 0.011743 | 4.21E-08 |
| rs61078946 | 16 | 75640517 | T | A | -0.08963 | 0.016194 | 3.12E-08 |
| rs117169628 | 16 | 89262657 | A | G | 0.10114 | 0.013651 | 1.27E-13 |
| rs117985867 | 17 | 1356644 | G | C | 0.097506 | 0.02134 | 4.90E-06 |
| rs3785632 | 17 | 15945608 | T | C | 0.048061 | 0.010078 | 1.85E-06 |
| rs63750417 | 17 | 44060775 | T | C | -0.09176 | 0.011487 | 1.37E-15 |
| rs11654648 | 17 | 45913653 | T | C | -0.10988 | 0.020763 | 1.21E-07 |
| rs56364001 | 17 | 71453941 | C | G | 0.067111 | 0.014478 | 3.56E-06 |
| rs28537779 | 18 | 20033052 | C | A | 0.060991 | 0.011829 | 2.52E-07 |
| rs147508500 | 18 | 50087827 | C | T | 0.38451 | 0.080471 | 1.77E-06 |
| rs5009257 | 18 | 60239967 | A | C | 0.050011 | 0.010891 | 4.39E-06 |
| rs12610495 | 19 | 4717672 | G | A | 0.14828 | 0.011043 | 4.21E-41 |
| rs78295726 | 19 | 10426512 | T | C | 0.073968 | 0.013044 | 1.42E-08 |
| rs45524632 | 19 | 10596988 | A | C | 0.22336 | 0.034592 | 1.07E-10 |
| rs189201949 | 19 | 43664483 | C | G | 0.29729 | 0.061278 | 1.23E-06 |
| rs492602 | 19 | 49206417 | G | A | -0.05504 | 0.009534 | 7.81E-09 |
| rs676314 | 19 | 50865535 | G | A | 0.077962 | 0.010055 | 8.95E-15 |
| rs117328378 | 19 | 56598183 | T | C | -0.14672 | 0.029786 | 8.40E-07 |
| rs2326788 | 20 | 6470094 | A | G | -0.05471 | 0.010484 | 1.80E-07 |
| rs1884826 | 20 | 19677714 | A | G | 0.062437 | 0.013058 | 1.74E-06 |
| rs6141933 | 20 | 31959411 | C | T | 0.09482 | 0.019904 | 1.90E-06 |
| rs9636867 | 21 | 34609944 | G | A | 0.12824 | 0.010176 | 2.05E-36 |
| rs78314212 | 21 | 35312916 | T | C | 0.122 | 0.017175 | 1.22E-12 |
| rs4822126 | 22 | 42792160 | C | G | -0.04723 | 0.010197 | 3.63E-06 |

Abbreviations: CHR, chromosome; ALT, effect allele; REF, other allele; SE, sebeta.
