# Supplementary material for: Causality between COVID-19 and multiple myeloma: a two-sample Mendelian randomization study and Bayesian co-localization
Source: Clin Exp Med. 2024 Feb 24;24(1):42. doi: 10.1007/s10238-024-01299-y (PMC10894079; doi:10.1007/s10238-024-01299-y)
Supplement: Supplementary file 3 — (docx 20 KB) [file 10238_2024_1299_MOESM3_ESM.docx]

Supplementary Table 3. IVs we selected for severe COVID-19.

| SNP | CHR | POS | ALT | REF | BETA | SE | *P* value |
| --- | --- | --- | --- | --- | --- | --- | --- |
| rs2505973 | 1 | 9122669 | G | A | -0.069274 | 0.014176 | 1.03E-06 |
| rs12407679 | 1 | 39578167 | T | C | -0.068504 | 0.014102 | 1.19E-06 |
| rs4660459 | 1 | 41254033 | C | T | 0.076101 | 0.015405 | 7.81E-07 |
| rs11208559 | 1 | 65435283 | G | C | 0.10253 | 0.017132 | 2.17E-09 |
| rs7528932 | 1 | 77949129 | T | A | 0.080198 | 0.014844 | 6.56E-08 |
| rs41264915 | 1 | 155167786 | G | A | -0.20626 | 0.022896 | 2.09E-19 |
| rs116415481 | 1 | 155286822 | T | C | 0.20909 | 0.041801 | 5.67E-07 |
| rs568035 | 1 | 156110167 | T | C | 0.14307 | 0.025967 | 3.60E-08 |
| rs860301 | 1 | 228287824 | C | T | -0.1339 | 0.027338 | 9.69E-07 |
| rs12614007 | 2 | 57316503 | A | G | 0.094133 | 0.016881 | 2.46E-08 |
| rs1123573 | 2 | 60707588 | G | A | -0.10621 | 0.0152 | 2.80E-12 |
| rs7588831 | 2 | 198508016 | G | A | 0.070989 | 0.014577 | 1.12E-06 |
| rs17279437 | 3 | 45814094 | A | G | -0.17214 | 0.025124 | 7.29E-12 |
| rs17713054 | 3 | 45859651 | A | G | 0.75584 | 0.026008 | 1.09E-185 |
| rs2593862 | 3 | 76581514 | G | A | 0.20768 | 0.044657 | 3.31E-06 |
| rs13094753 | 3 | 101525082 | G | A | -0.071324 | 0.01509 | 2.28E-06 |
| rs343320 | 3 | 146234909 | A | G | 0.15423 | 0.027508 | 2.06E-08 |
| rs9819533 | 3 | 168790718 | A | G | 0.11811 | 0.023728 | 6.44E-07 |
| rs4955646 | 3 | 169118180 | G | C | 0.10304 | 0.022147 | 3.28E-06 |
| rs150285670 | 3 | 172465616 | A | C | -0.32212 | 0.070005 | 4.20E-06 |
| rs6765278 | 3 | 188004464 | A | G | 0.07575 | 0.015921 | 1.96E-06 |
| rs75410063 | 4 | 7879049 | C | T | 0.089503 | 0.018409 | 1.16E-06 |
| rs7664615 | 4 | 25448493 | G | A | -0.097683 | 0.019378 | 4.63E-07 |
| rs34712979 | 4 | 106819053 | A | G | -0.10951 | 0.01701 | 1.21E-10 |
| rs11131812 | 4 | 168686778 | A | G | 0.076121 | 0.014285 | 9.90E-08 |
| rs4956966 | 5 | 732378 | A | G | -0.13091 | 0.02698 | 1.22E-06 |
| rs1874166 | 5 | 28065778 | T | A | 0.082352 | 0.015537 | 1.16E-07 |
| rs10066378 | 5 | 131776967 | C | T | 0.11796 | 0.021006 | 1.96E-08 |
| rs1128175 | 6 | 31150435 | G | A | -0.1255 | 0.017115 | 2.25E-13 |
| rs4711304 | 6 | 32660170 | C | T | -0.068659 | 0.01382 | 6.76E-07 |
| rs41435745 | 6 | 41490382 | C | G | 0.26369 | 0.052723 | 5.69E-07 |
| rs13212511 | 6 | 73669925 | G | A | 0.17883 | 0.038186 | 2.83E-06 |
| rs76579458 | 6 | 99084947 | C | T | 0.51756 | 0.11288 | 4.54E-06 |
| rs9505716 | 6 | 115339550 | T | C | 0.11786 | 0.025616 | 4.21E-06 |
| rs72993537 | 6 | 145550399 | T | C | 0.19939 | 0.041123 | 1.24E-06 |
| rs60220083 | 7 | 22838314 | T | C | -0.12726 | 0.02722 | 2.94E-06 |
| rs7785991 | 7 | 25700211 | T | C | -0.084244 | 0.01801 | 2.90E-06 |
| rs12534422 | 7 | 75263792 | T | C | 0.085624 | 0.015073 | 1.34E-08 |
| rs2897075 | 7 | 99630342 | T | C | 0.087987 | 0.014375 | 9.32E-10 |
| rs737045 | 8 | 26724077 | A | G | 0.15919 | 0.034402 | 3.70E-06 |
| rs2625446 | 8 | 61540398 | A | G | 0.067381 | 0.014353 | 2.67E-06 |
| rs77264633 | 9 | 15823918 | G | A | 0.1209 | 0.026197 | 3.93E-06 |
| rs28368148 | 9 | 21206605 | G | C | 0.44878 | 0.065493 | 7.26E-12 |
| rs10978600 | 9 | 109467965 | A | T | 0.077084 | 0.015431 | 5.87E-07 |
| rs550057 | 9 | 136146597 | T | C | 0.11703 | 0.015941 | 2.12E-13 |
| rs17885848 | 10 | 81316456 | T | C | 0.090196 | 0.015623 | 7.78E-09 |
| rs12259804 | 10 | 115573378 | C | T | -0.23866 | 0.048809 | 1.01E-06 |
| rs35705950 | 11 | 1241221 | T | G | -0.1636 | 0.022785 | 6.95E-13 |
| rs61882275 | 11 | 34504292 | A | G | -0.12605 | 0.014659 | 8.08E-18 |
| rs71481218 | 11 | 49931219 | A | T | -0.21719 | 0.045394 | 1.71E-06 |
| rs75230921 | 11 | 134152143 | A | G | 0.16315 | 0.034991 | 3.12E-06 |
| rs75505823 | 12 | 57540956 | T | G | -0.2126 | 0.044922 | 2.22E-06 |
| rs10850097 | 12 | 113361117 | T | C | 0.095247 | 0.014916 | 1.71E-10 |
| rs11614702 | 12 | 133058157 | A | G | 0.10135 | 0.01383 | 2.33E-13 |
| rs12585036 | 13 | 113535741 | T | C | 0.14143 | 0.017239 | 2.32E-16 |
| rs10459574 | 14 | 104083042 | G | C | 0.082664 | 0.016353 | 4.31E-07 |
| rs151219844 | 15 | 27693038 | T | C | 0.31637 | 0.069096 | 4.68E-06 |
| rs2102497 | 16 | 54255222 | C | T | 0.078907 | 0.017145 | 4.18E-06 |
| rs117169628 | 16 | 89262657 | A | G | 0.15749 | 0.020078 | 4.36E-15 |
| rs3785632 | 17 | 15945608 | T | C | 0.067635 | 0.014796 | 4.85E-06 |
| rs57708672 | 17 | 33571309 | T | G | -0.090779 | 0.019649 | 3.84E-06 |
| rs6503533 | 17 | 38184580 | T | C | 0.069068 | 0.014723 | 2.72E-06 |
| rs62056905 | 17 | 43782693 | G | A | -0.1285 | 0.016821 | 2.18E-14 |
| rs112698697 | 17 | 63938142 | A | G | -0.25744 | 0.054333 | 2.16E-06 |
| rs4800403 | 18 | 20016089 | T | C | 0.081109 | 0.017683 | 4.50E-06 |
| rs12610495 | 19 | 4717672 | G | A | 0.24167 | 0.016049 | 3.05E-51 |
| rs2569703 | 19 | 10404227 | G | C | -0.10827 | 0.014048 | 1.28E-14 |
| rs368565 | 19 | 49201217 | T | C | 0.1056 | 0.014909 | 1.41E-12 |
| rs60132559 | 19 | 50867106 | T | C | 0.091456 | 0.015009 | 1.11E-09 |
| rs2326788 | 20 | 6470094 | A | G | -0.08057 | 0.015432 | 1.78E-07 |
| rs9636867 | 21 | 34609944 | G | A | 0.18394 | 0.015088 | 3.47E-34 |
| rs7279933 | 21 | 34672515 | C | T | -0.068109 | 0.01421 | 1.64E-06 |
| rs2236645 | 21 | 35342238 | T | C | 0.17941 | 0.024951 | 6.46E-13 |
| rs2070788 | 21 | 42841988 | A | G | -0.07029 | 0.014472 | 1.19E-06 |

Abbreviations: CHR, chromosome; ALT, effect allele; REF, other allele; SE, sebeta.
