# Supplementary material for: Causality between COVID-19 and multiple myeloma: a two-sample Mendelian randomization study and Bayesian co-localization
Source: Clin Exp Med. 2024 Feb 24;24(1):42. doi: 10.1007/s10238-024-01299-y (PMC10894079; doi:10.1007/s10238-024-01299-y)
Supplement: Supplementary file 4 — (docx 14 KB) [file 10238_2024_1299_MOESM4_ESM.docx]

Supplementary Table 4. IVs we selected for MM.

| SNP | CHR | POS | ALT | REF | BETA | SE | *P* value |
| --- | --- | --- | --- | --- | --- | --- | --- |
| rs75358643 | 4 | 160448221 | G | A | 1.75 | 0.333 | 1.48E-07 |
| rs78782621 | 8 | 116203709 | C | T | 1.2687 | 0.2759 | 4.27E-06 |

Abbreviations: CHR, chromosome; ALT, effect allele; REF, other allele; SE, sebeta.
